# Identification of an immunodominant IgE epitope of Der p 39, a novel allergen of *Dermatophagoides pteronyssinus*

**DOI:** 10.1016/j.waojou.2022.100651

**Published:** 2022-05-06

**Authors:** Wei-Yong Li, Ze-Lang Cai, Bo-Ping Zhang, Jia-Jie Chen, Kunmei Ji

**Affiliations:** Department of Biochemistry and Molecular Biology, School of Basic Medical Sciences, Health Science Center, Shenzhen University, No. 1066 Xueyuan Road, Nanshan District, Shenzhen 518060, China

**Keywords:** HDM, Der p 39, Immunodominant IgE epitope, IgE-binding

## Abstract

**Background:**

House dust mites (HDMs) are the main source of indoor inhalatory allergens that cause IgE-mediated allergic diseases. The discovery and identification of HDM allergens are important for the diagnosis and treatment of allergic diseases.

**Objective:**

We sought to identify a Group 39 *Dermatophagoides pteronyssinus* (Der p) allergen, namely Der p 39, and explore its immunodominant IgE epitopes.

**Methods:**

Homology analysis of amino acid (aa) sequences in HDM and human troponin C (TnC)-like protein was performed. Total RNA of Der p was extracted and used to amplify Der p 39 cDNA with specific primers. Recombinant Der p 39 protein was expressed with a pET-His prokaryotic expression system and purified with Ni-NTA resins. IgE binding was evaluated with western blot, dot blot, and enzyme-linked immunosorbent assay (ELISA) experiments. The IgE binding epitopes of Der p 39 were identified by observing HDM-allergic sera interactions with truncated and hybrid proteins formed from Der p 39 and human TnC-like proteins.

**Results:**

The Der p 39 open reading frame (ORF) cDNA was found to be 462 base pairs and registered in the NCBI library (GenBank no. MZ336019.1). Der p 39, which encoded 153 aa, was found to have 35.63% and 99.35% homology with human TnC and *Dermatophagoides farina* (Der f) 39, respectively. IgE-ELISA showed IgE binding with expressed and purified recombinant Der p 39 (18 kDa) in 5/87 (5.75%) HDM-allergic sera samples. Analyses of IgE binding with Der p 39-based truncated and hybrid proteins indicated that IgE binding epitopes are likely located in the C-terminal region and dependent on conformational structure. The data from this study were submitted to the World Health Organization and International Union of Immunological Societies (WHO/IUIS) Allergen Nomenclature database.

**Conclusion:**

Der p 39 was identified as a minor HDM allergen with a conformational IgE binding epitope. These findings could have important theoretical implications in the development of HDM allergy diagnostics and therapeutics.

## Introduction

House dust mite (HDM) allergens are a major cause of human allergic diseases, including allergic rhinitis, allergic asthma, specific rhinitis, and other allergic skin diseases.^1^, ^2^, ^3^, ^4^, ^5^ The World Health Organization (WHO) estimated that 600 million people worldwide suffer from allergic rhinitis, of which 200 million experience asthma.^6^^,^^7^ Worldwide, the most prominent indoor mites are *Dermatophagoides pteronyssinus* (Der p) and *Dermatophagoides farinae* (Der f), which are ubiquitous in dust samples from temperate or tropical regions.^8^, ^9^, ^10^, ^11^

The identification of HDM allergens has direct guiding significance for the diagnosis and treatment of HDM-induced allergic diseases. HDM allergens induce allergic immune responses by binding serum IgEs.^12^ However, it is not yet known how many HDM allergens there are; and the work of sequencing and determining the biochemical characteristics and structures of novel HDM allergens is ongoing.^13^ At present, 39 HDM allergen groups have been reported, though the WHO/IUIS (International Union of Immunological Societies) allergen database identifies only 31 Der p allergen groups and 36 Der f allergen groups.^14^ Previously, based on transcriptome analyses and proteome analyses of the HDM allergome, we reported that the group 24 HDM allergens, namely Der f 24 and Der p 24, were UQCRB (ubiquinol cytochrome C reductase binding protein) homologs.^15^^,^^16^ Recently, amino acid (aa)-sequence homology comparisons with the chromosome-level Der f genome and transcriptome revealed three HDM allergens, namely Der f 23, Der f 37, and Der f 39.^17^ Der f 39 is a TnC-like protein that has 95.42% homology with the storage mite allergen Tyr p 34 and binds with serum IgEs in enzyme-linked immunosorbent assay (ELISA) (positive rate: 9.21%, 7/76).^17^^,^^18^ However, a Group 39 allergen has not yet been identified in Der f.

Allergenicity is established with the confirmation IgE binding activity.^19^^,^^20^ The specific recognition of the IgE binding epitope (a.k.a. B cell epitope) on the allergen by IgE antibodies bound to effector cells such as basophils and mast cells is essential for the development of the allergic response.^21^ The formation of cross-linked complexes between these epitopes and mast cell surface receptors induce allergic inflammation.^22^ Thus IgE binding epitopes can be used in allergic disease diagnosis and they have the potential to inform the development of an HDM vaccine.^23^ Up to now, only IgE binding epitopes for Group 1, 2, 3, 7, 11, 13, 23, 24, and 33 HDM allergens have been confirmed empirically.^15^^,^^24^, ^25^, ^26^, ^27^, ^28^, ^29^, ^30^, ^31^, ^32^ An immunodominant IgE epitope of Der p 39 remains to be established.

The aim of this study was to identify whether Der p 39 is a novel HDM allergen. We cloned, expressed, and purified Der p 39 recombinant protein. We subjected the resultant recombinant Der p 39 to serum IgE binding assays and used IgE binding experiments, including western blots, dot blots, and ELISAs, to identify the immunodominant IgE epitope of Der p 39. The thus characterized novel Der p 39 allergen was submitted to the WHO/IUIS database.

## Methods

### Patients' sera

Informed consent was obtained from all patients and volunteers. The protocol of the study was approved by the Ethics Committee of the First Affiliated Hospital of Guangzhou Medical College. Sera from 87 patients with HDM allergies and control sera from 30 healthy volunteers were obtained at Peking Union Medical College Hospital (Beijing, China). The HDM allergic group consisted of patients who had experienced HDM-triggered anaphylaxis or had IgE levels higher than 3 in ImmunoCAP allergen detection system testing (Pharmacia Diagnostics, Uppsala, Sweden). Their allergic responses were confirmed by clinical history and diagnosis, and then characterized by measuring specific IgE reactivity with an ImmunoCAP allergen detection system (Table S1). All procedures involving human participants were conducted in accordance with the ethical standards of the committee.

### Gene cloning

Total RNA was isolated from HDMs with an Minibest Universal RNA Extraction Kit (TaKaRa, Tokyo, Japan) and reverse transcribed rapidly into cDNAs with an Evo M-MLV RT Mix Kit (Accurate Biology, Changsha, China). Der p 39-specific primers were designed based on the results of the Der p genome (NCBI Genome ID 8901). The forward primer was 5′-CTTATCCAAAATGTCTGTCG-3′ and the reverse primer was 5′-TGTAACGTTTTTAATCACCA-3’. Der p 39 open reading frame (ORF) DNA was amplified by polymerase chain reaction (PCR) and analyzed by agarose gel electrophoresis. The product of which was cloned into a pMD 19-T vector (TakaRa, Dalian, China). The nucleotide sequence of the cloned product was determined by DNA sequencing.

### Homologous comparison of aa sequences

The aa sequences of Der p 39 (GenBank accession no. MZ336019.1), Der f 39 (GenBank accession no. MK419032.1), and human TnC (GenBank accession no. M22307.1) were saved in FASTA format and aligned in DNAMAN 8 (version 8.0; Lynnon Biosoft).

### Identification of native Der p 39 protein by mass spectrometry (MS)

To extract total protein, HDM bodies were ground up with liquid nitrogen. The pulverized HDM tissue was lysed in RIPA buffer containing protease inhibitor cocktail (MedChem Express, Monmouth Junction, NJ). The lysate was then broken up with ultrasonic disruption and centrifuged at 8000×*g* for 30 min. The supernatant was subjected to protein concentration detection and sodium dodecyl-sulfate polyacrylamide gel electrophoresis (SDS-PAGE). A protein band with a molecular weight of ∼18-kDa was cut out and digested in trypsin. The digested polypeptide samples were analyzed by liquid chromatography-MS (Q Exactive, ThermoFisher, USA).

### Expression and purification of recombinant Der p 39 and related proteins

An artificial codon-optimized Der p 39 sequence (GenBank no. MZ643463.1) was synthesized by Genescript Corporation (Nanjing, China) and subcloned into the prokaryotic expression vector pET-His (miaolingbio, Wuhan, China). The resultant recombinant plasmid pET-His-Der p 39 was transformed into *Escherichia coli* BL21 (DE3) pLysS cells and grown in Luria-Bertani medium containing 10 mg/mL kanamycin. After 3 h of growth at 37 °C 1-mM isopropyl ß-d-1-thiogalactopyranoside (IPTG)-induced expression was observed and cells were harvested by centrifugation at 8000×*g* for 2 min. Pellets were resuspended in buffer (20 mM Tris-HCl, 150 mM NaCl, pH 8.0) and the cells were lysed by ultrasonic homogenization. The supernatant and precipitate were collected and analyzed by SDS-PAGE. The recombinant Der p 39 protein was expressed as a soluble protein and purified by Ni-NTA gel affinity chromatography (GE Healthcare, USA).

Five specific truncated forms of Der p 39 were used to screen for IgE epitopes. The cDNA sequences encoding truncated-form proteins were synthesized by Genescript Corporation (Nanjing, China) and subcloned into pET-DsbA prokaryotic expression vectors (miaolingbio, Wuhan, China) for recombinant protein expression. The truncated forms of Der p 39 were employed, namely Der p 39 T1 (1–50 aa), Der p 39 T2 (31–80 aa), Der p 39 T3 (61–110 aa), Der p 39 T4 (91–140 aa), and Der p 39 T5 (141–153 aa). In addition, hybrid proteins that integrated Der p and human TnC protein sequences into Der p 39 were designed; the truncated and hybrid Der p 39 cDNA sequences were synthesized into proteins by Genescript Corporation (Nanjing, China). The artificial synthetic genes Der p 39-Hyb1, Der p 39-Hyb2, Der p 39-Hyb3, Der p 39-Hyb4, Der p 39-Hyb5, and Der p 39-Hyb6 were registered in the GenBank (accession no. MZ465537.1, MZ465538.1, MZ465539.1, MZ465540.1, MZ465541.1, and MZ465542.1, respectively). These cDNAs were subcloned into pET-His vectors. The expression and purification of recombinant protein was performed as described above. The purified recombinant proteins’ concentrations were measured with Bradford assays (Sangon Biotech Co., Ltd).

### IgE-ELISA

The IgE-ELISA assay developed in this study was performed as previously described.^33^ Microtiter plates were coated with recombinant polypeptides (200 ng/well) in 0.1 mol/L and pH 9.2 carbonate buffered solution (Leagene, Beijing, China) at 4 °C overnight. The samples were blocked with 300 μl 5% (w/v) Difco™ skim milk (DSM; BD Biosciences) in phosphate-buffered saline containing 0.05% Tween 20 (PBST) at 37 °C for 3 h. Serum samples (1:10 dilution in 1% DSM-PBST) were added to each well (100 μL/well) and incubated for 2.5 h at 37 °C. The plates were incubated with mouse anti-human IgE horseradish peroxidase-conjugated antibody (#9160-05; 1:2000 dilution; Southern Biotech) for 1.5 h at 37 °C. Each incubation step was followed by five washes with PBST. Binding was detected with 100 μl of 1-mM 3,3,5,5′-tetramethylbenzidine substrate (Invitrogen; Thermo Fisher Scientific); the substrate reaction was stopped with 50 μl 2 M H_2_SO_4_ per well. The plates were read by an absorbance microplate reader (Bio-Rad, USA) at 450 nm. IgE-ELISA results with a positive/negative result sample optical density ratio value > 2.1 were considered positive. All tests were performed in triplicate.

### IgE-western blot

Antibody binding with purified proteins was determined by IgE western blot assays, performed as described previously.^33^ Briefly, proteins were separated by 12% SDS-PAGE and transferred to polyvinylidene-fluoride membranes (Millipore, USA). The membranes were washed three times (5 min each) with Tris Buffered Saline containing 0.05% Tween 20 (TBST), blocked with blocking buffer (TBST with 5% DSM) at 4 °C overnight, washed three times (10 min each) with TBST, and then incubated with serum samples (1:10 dilution in 1% DSM-TBST) for 2.5 h at 37 °C. After washing three times (10 min each time) with TBST, the serum-bound membranes were incubated in TBST containing 1% DSM and HRP-conjugated mouse anti-human IgE antibody (#9160-05; 1:2000 dilution; Southern Biotech) for 1.5 h at 37 °C. After washing again in TBST, bands were visualized with a Pierce™ 3′-diaminobenzidine substrate kit (Thermo Fisher Scientific). The reaction was stopped with TBST or double-distilled water.

### IgE-dot blot

The binding affinities of IgE antibodies with purified proteins were determined by IgE dot blot assays, performed as described previously.^33^ Briefly, 1 μl of 2 mg/mL aliquots of peptides were spotted serially onto nitrocellulose membrane (Millipore, USA) and left to dry for 30 min at room temperature. The spotted membranes were blocked with blocking buffer (TBST with 5% DSM) at 4 C° overnight. After three washes with PBST (10 min each), membranes were incubated with serum samples (1:10 dilution in 1% DSM-TBST) for 2 h at 37 °C, washed three times (10 min each), and incubated in TBST containing 1% DSM with HRP-labeled mouse anti-human IgE antibody (#9160-05; 1:2000 dilution; Southern Biotech) for 1 h at 37 °C. After three washes with TBST, the membranes were incubated with 3′-diaminobenzidine substrate for 3 min. The reaction was stopped and with TBST or double-distilled water.

### Structure modeling of Der p 39

The secondary structure of Der p 39 was analyzed with an online tool called Sopma Secondary Structure Prediction Method, which is available at https://npsa-prabi.ibcp.fr/cgi-bin/npsa_automat.pl?page=npsa_sopma.html. A *Lethocerus indicus* TnC structural template was obtained from the RCSB Protein Data Bank (http://www.rcsb.org/; PDB code, 2JNF.1) and used for three-dimensional (3D) molecular modeling of Der p 39. We generated a 3D model structure and associated rendered images in SWISS-MODEL software in the SWISS-MODEL server.

### Statistical analysis

All data were analyzed in Prism 7 (GraphPad Software, Inc.) and expressed as means ± standard deviations. Differences between the allergic and control groups were determined by one-way analyses of variance (ANOVAs) followed by Dunnett's post-hoc tests for multiple comparisons. Each treatment was examined in triplicate and *p* < 0.05 was considered to indicate a statistically significant difference.

### Data availability

All data described in this study are available in this manuscript or a supporting information file. Submitted allergen data are available on the WHO/IUIS allergen nomenclature database (http://www.allergen.org/viewallergen.php?aid=1070).

## Results

### Gene cloning and protein identification of Der p 39

**Fig. 1:**
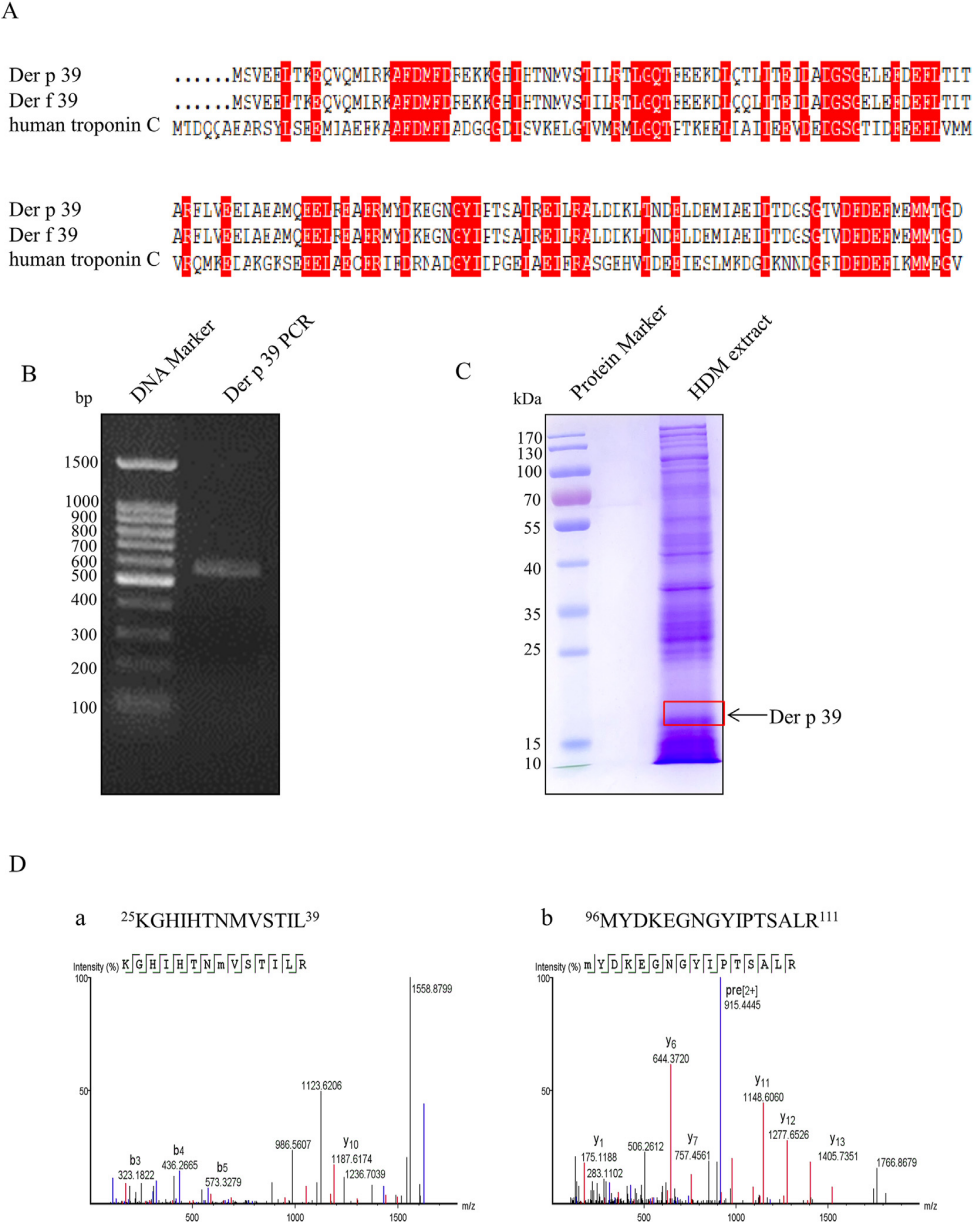
Gene cloning and protein identification of Der p 39. A. Comparison of Der f 39, Der f 39, and human TnC aa sequences. Homologous sequences are shaded in red. B. Der p 39 cDNA was amplified with specific primers and confirmed by Sanger sequencing. C. SDS-PAGE analysis of crude Der p protein extract stained with Coomassie brilliant blue. D. MS analysis showing peptide coverage of natural Der p 39 (protein band found at ∼18 kDa); data for the 26–39 aa peptide (a) and the 96–111 aa peptide (b) are shown.

The homology of the aa sequence of a TnC-like protein in Der p (NCBI Genome ID 8901) with the aa sequences of Der f 39 and human TnC-like protein are shown in Fig. 1A. Der p TnC-like protein was found to be 99.35% homologous with Der f 39 and to have a single aa difference at position 52. The Der p 39 ORF sequence gene was cloned from the Der p cDNA library and verified by DNA sequencing to match the predicted Der p genome (Fig. 1B). The ORF cDNA sequence of Der p 39 and its sequence was registered in the NCBI library (GenBank No. MZ336019.1); it is 462 base pairs and encodes 153 aa with a theoretical molecular mass of 18 kDa. We thus extracted an ∼18-kDa protein band from an SDS-PAGE gel of Der p crude extract and subjected its contents to liquid chromatography-MS analysis to identify the putative endogenous Der p 39 protein (Fig. 1C). MS analysis indicated that a 61.0% coverage rate was achieved. Representative results from two coverage peptides (26–39 aa and 96–111 aa) are shown in Fig. 1D. These data confirm that Der f 39 homologue TnC-like protein was present in Der p.

### Identification of Der p 39 as a novel HDM allergen

**Fig. 2:**
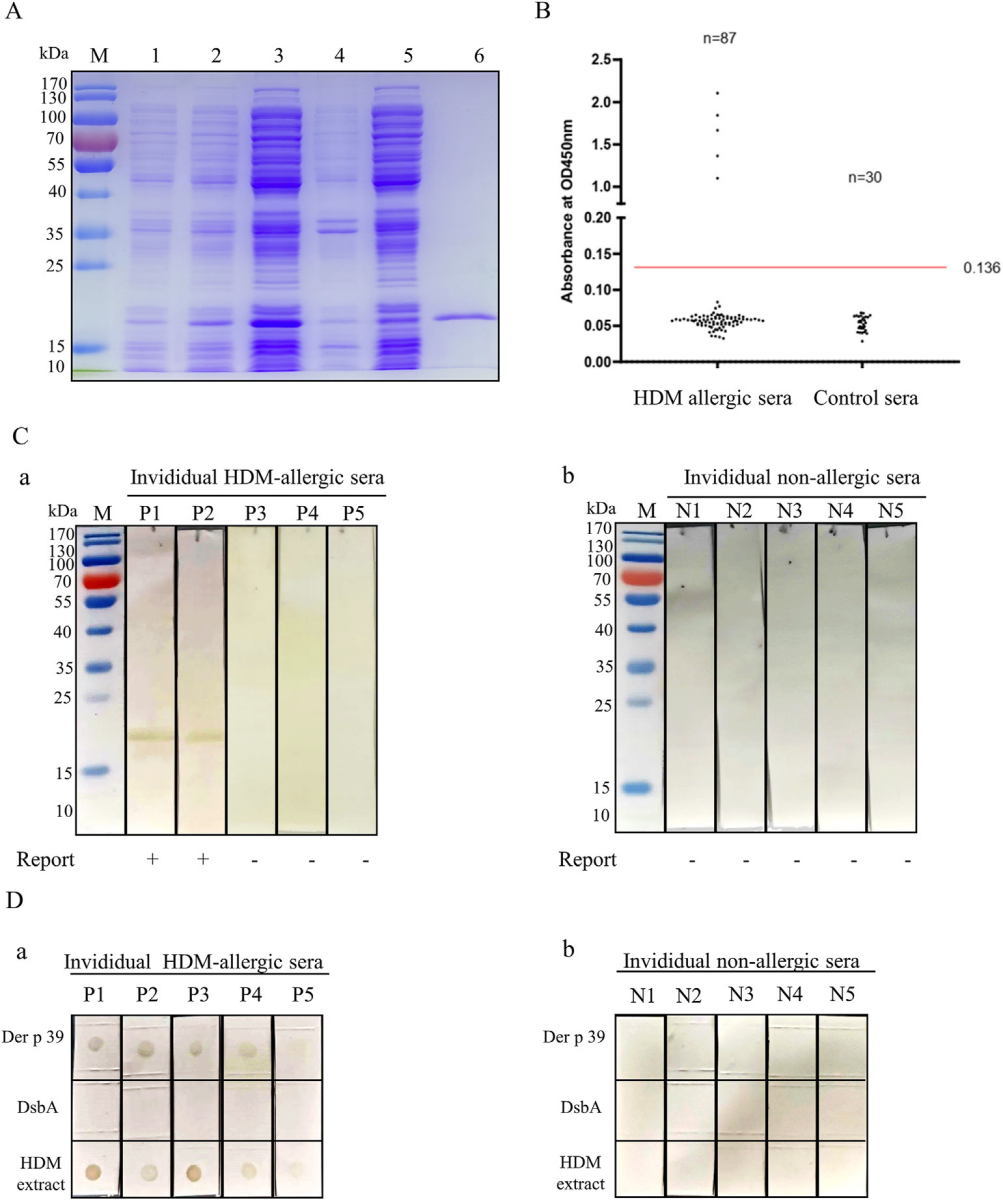
*In vitro* IgE binding reactivity of Der p 39. A. SDS-PAGE of purified Der p 39 from *E. coli* BL21 (DE3) cells transformed with pET-His-Der p 39 plasmid stained with Coomassie brilliant blue. Lane M: marker. Lane 1/2: *E. coli* transformed with pET-His-Der p 39 plasmid before/after IPTG induction. Lane 3/4: ultrasonicated supernatant/sediment from IPTG-induced *E. coli* transformed with pET-His-Der p 39. Lane 5: Ni column flow-through. Lane 6, purified recombinant Der p 39 protein. B. IgE-ELISA of serum IgE reactivity to Der p 39 show IgE binding to recombinant Der p 39 protein in 5/87 (5.75%) HDM-allergic sera samples (vs. 30 non-allergic controls); positive result optical density cut-off value of 0.136. Western blot (C) and dot-blot (D) analyses of Der p 39 reactivity with individual HDM-allergic (a) and non-allergic (b) patient serum samples (5 individuals per group).

To obtain Der p 39 protein, we constructed a prokaryotic pET-His vector with an artificial codon-optimized sequence (GenBank No. MZ643463.1) and transformed the vector into *E. coli* for expression and purification. After ultrasonication, IPTG induction, Ni-NTA-resin purification, SDS-PAGE analysis confirmed that 18-kDa Der p 39 was expressed in the supernatant (Fig. 2A). IgE-ELISA showed that 5/87 patients with HDM allergies (5.75%) had specific IgE activity binding to Der p 39. None of the serum samples from 30 non-allergic individuals were bound by IgE (Fig. 2B). IgE western blot (Fig. 2C) and dot blot (Fig. 2D) assays showed that Der p 39 protein bound selectively by HDM allergic serum IgEs but not non-allergic serum. Based on the above results indicating that Der p 39 acts as an allergen, Der p 39 has been added to the WHO/IUIS Allergen Nomenclature database (http://www.allergen.org/viewallergen.php?aid=1070).

### Immunodominant IgE epitopes of Der p 39

**Fig. 3:**
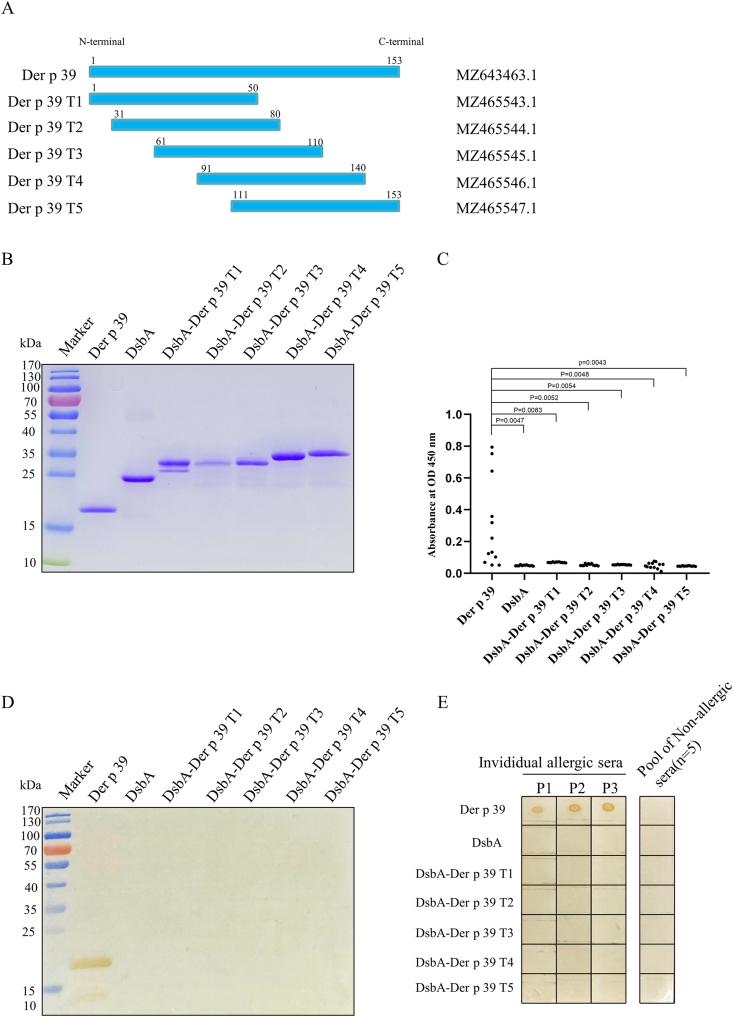
Screening of truncated Der p 39 proteins for immunodominant IgE binding. A. Schematic diagram of five produced truncated proteins. B. SDS-PAGE analysis of purified Der p 39, truncated Der p 39-DsbA fusion proteins, and DsbA with Coomassie brilliant blue staining. C. IgE-ELISA of Der p 39, truncated Der p 39-DsbA fusion proteins, and DsbA with HDM allergic sera from 12 individuals. D. Western blot assay of IgE binding capacity with allergic sera pooled from 5 HDM allergic individuals (from IgE-ELISA positive samples). E. Dot blot assay of IgE binding capacity with HDM allergic sera from 3 individuals and non-allergic sera from 5 individuals.

Five truncated Der p 39 proteins were separated by SDS-PAGE of purified proteins obtained from pET-DsbA vector-expressed truncated Der p 39 cDNAs (Fig. 3Aand B): Der p 39 T1 (1–50 aa), Der p 39 T2 (31–80 aa), and Der p 39 T3 (61–110 aa), Der p 39 T4 (91–140 aa), and Der p 39 T5 (141–153 aa). According to IgE-ELISA (Fig. 3C), western blot (Fig. 3D), and dot blot (Fig. 3E) analyses, none of these five truncated proteins were reactive with IgEs from Der p 39-binding sera, indicating that the immunodominant IgE epitope(s) of Der p 39 may be conformational.

### Localization of immunodominant IgE epitopes of Der p 39

**Fig. 4:**
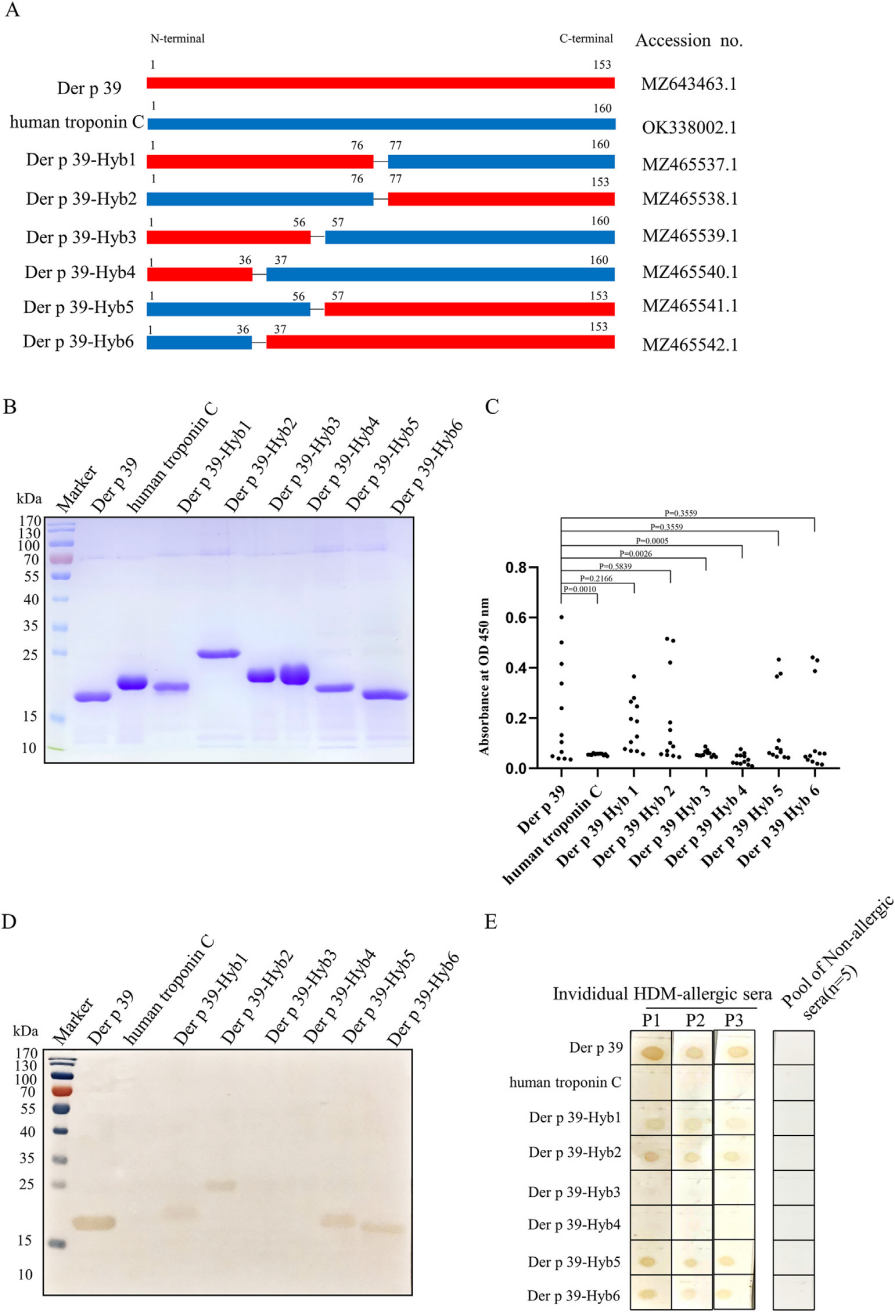
IgE binding epitope detection analysis with Der p 39 hybrid proteins. A. Schematic diagram of Der p 39 and hybrid proteins. B. SDS-PAGE of purified Der p 39, human TnC, and Der p 39-hybrid proteins with Coomassie brilliant blue staining. C. IgE-ELISA showing IgE binding of human TnC and Der p 39-hybrid proteins with 8 individual HDM allergic sera. D. Western blot assay of IgE binding with pooled serum from 5 HDM allergic (i.e., IgE-ELISA positive) individuals. E. Dot blot assay of IgE binding with individual serum samples from 3 HDM allergic individuals and pooled serum from 5 non-HDM allergic individuals.

**Fig. 5:**
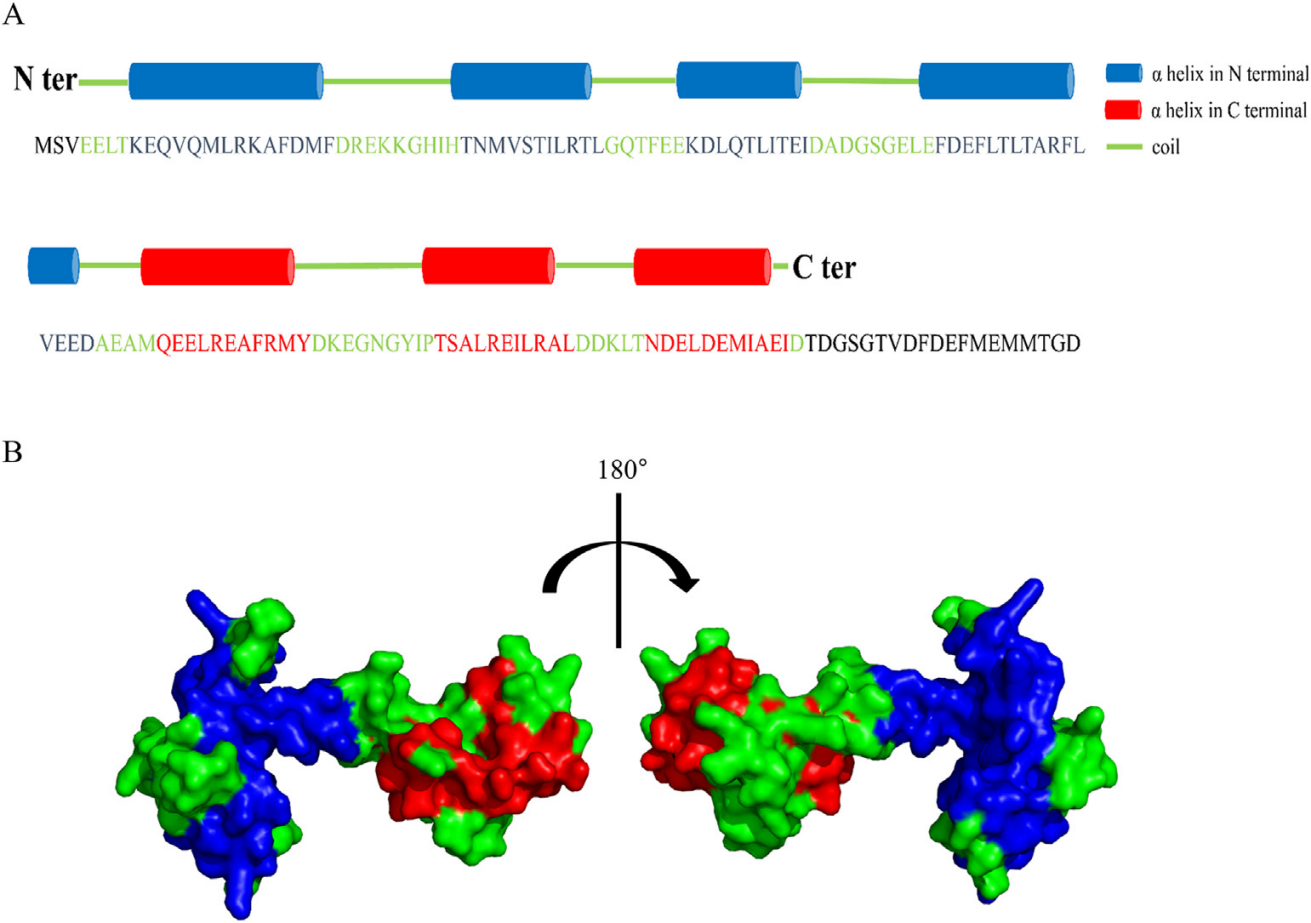
Structural analysis and modelling of Der p 39. A. Schematic representation showing location of potential epitopes on the secondary structure of Der p 39. B. Homology model of Der p 39 based on crystal structure template from *L. indicus* TnC (PDB code, 2JNF.1) indicating potential epitope sites

Given the aforementioned supposition that the immunodominant IgE epitope(s) of Der p 39 may be formed by a 3D spatial structure, we employed the human TnC protein (a Der p 39 homolog) to assist in localizing likely immunodominant IgE epitope components (Fig. 1A). The Der p and human components of purified recombinant Der p 39, hybrid Der p 39 proteins, and human TnC protein are delineated in Fig. 4A. Hybrid proteins were expressed in a pET-His expression system, purified with Ni-NTA resins, and analyzed by SDS-PAGE (Fig. 4B). IgE-binding assays showed that three hybrid proteins (Der p 39-Hyb2, Der p 39-Hyb5, and Der p 39-Hyb6) had significantly more IgE binding ability than the others (Fig. 4C–E). Secondary structure analysis showed that the C-terminal region of Der p 39 had three potential antigen-binding epitopes: E5 (^87^QEELREAFRMY^97^), E6 (^107^TSALREILRAL^117^), and E7 (^123^NDELDEMIAEI^133^) (Fig. 5A). Homologous 3D structural modeling in SWISS-MODEL software indicated that the C-terminal region of Der p 39 has three α-helices positioned on the outside of the whole folded protein (Fig. 5B). This external location may facilitate IgE binding. These data indicate that the immunodominant IgE epitope(s) of Der p 39 may be formed by aa spans in the C-terminal region.

## Discussion

The present experiments showed that the ORF cDNA of Der p 39 is 462 base pairs long (GenBank no. MZ336019.1) and encodes 153 aa with a theoretical molecular mass of 18 kDa. We showed that Der p 39 has 35.63% and 99.35% homology with human TnC and Der f 39, respectively. Our purified recombinant Der p 39 (∼18 kDa) reacted with 5/87 HDM-allergic serum samples. Our binding experiments with Der p 39-based truncated and hybrid proteins indicated that the immunodominant IgE binding epitope or epitopes of Der p 39 are likely to be conformational and may be located in the C-terminal region.

Although Der p and Der f allergen components are generally highly homologous,^14^ the allergenicity of same-allergen-group proteins from these two species may differ. For example, Der p 23 and Der f 23 react with 74.0% and 55.8% of HDM allergic patient sera, respectively.^34^^,^^35^ The known major HDM allergens do not account for all reactions of HDM allergic sera. Together, the major allergens Der f 1 and Der f 2 react with only 95.8% of HDM allergic sera.^36^ HDM minor allergens with low IgE-binding ability, such as Group 5 and Group 10 allergens, can also promote allergic inflammation. Therefore, there remains a need to identify novel HDM allergens.^37^^,^^38^

Notably, we found that the aa sequence of Der p 39 is a TnC-like protein that has 99.35% homology with the aa sequence of the previously identified minor HDM allergen Der f 39, with the two homologs differing by a single aa.^17^ The presently observed 5.75% (5/87) positive specific-IgE reaction rate observed for Der p 39 in this study was similar to the previously reported low positive rate for Der f 39 (9.21%; 7/76).^17^^,^^18^ Here, there was a limitation, lack of functional assay to show the IgE reactivity of Der p 39, such as histamine release assay, basophil activation test (BAT) or skin prick test (SPT).

Allergen IgE-binding activity depends on an IgE binding epitope. Previously, we found that the immunodominant IgE epitopes of HDM Group 24 allergens are located in the N-terminal region with a linear and conformational epitope structure.^15^^,^^16^ The present results suggest that the immunodominant IgE epitope of Der p 39 is formed by aa spans in the C-terminal region and dependent mainly on 3D conformational structure. Detailed knowledge of IgE and T cell epitopes of each of allergen molecule is crucial for the development of molecular allergy vaccines.^39^ Furthermore, the identification of immunodominant IgE epitopes of allergens can help to elucidate allergen-induced sensitization mechanisms and provide information that can be used to improve immunotherapy outcomes.^40^ For example, a Der p 2-mutant gene vaccine in which an IgE epitope was deleted has been reported to inhibit airway inflammation.^41^^,^^42^ Most HDM allergens have yet to be clearly resolved. Prior to this study, the B cell epitopes of Group 1, 2, 3, 7, 11, 13, 23, 24, and 33 HDM allergens had been identified.

It is difficult to identify a conformational epitope with sequential overlapping peptides methods. Previously, sequential Der p 7 peptides were reported to lack IgE reactivity.^43^ Similarly, none of the five truncated Der p 39 forms examined sequentially in this study showed IgE reactivity with HDM allergic sera, leading us to infer that the immunodominant IgE epitopes of Der p 39 is likely to be conformational. Indeed, several previously identified IgE binding epitopes of HDM allergens (Der p 1, Der p 2, Der p 7, and Der f 23) have been conformational.^35^^,^^43^, ^44^, ^45^ Generally, screening for allergen epitope regions of conformational IgE epitopes requires IgE antibodies derived from allergic patient-derived sera or artificially prepared monoclonal antibodies, which are time-consuming to collect or prepare, respectively.^46^ To circumvent this challenge, we employed Der p 39-based hybrid proteins and found that the IgE-binding epitope of Der p 39 is likely formed by the 3D conformational structure of the C-terminal region of the allergen. Using the same method previously, we found that the immunodominant IgE binding epitopes of Der f 24 were located in the N-terminal region of Der f 24.^16^

HDM Group 39 allergens belong to TnC family of proteins. TnC, a muscle related protein and member of the Ca^2+^ receptor protein calmodulin superfamily, plays an important regulatory role in muscle contraction and relaxation.^47^ In the future, it will be prudent to determine how, mechanistically, Der p 39 affects B-cell epitopes and whether Der p 39-induced allergic inflammation involves the activation of macrophages, epithelial cells, or other functional cells.

## Conclusion

The novel HDM allergen Der p 39 was identified. The immunodominant IgE binding epitope of Der p 39 appears to be conformational and located mainly in the C-terminal region of the allergen. This study can provide reference information relevant for HDM allergy diagnosis and specific immunotherapy development.

## Abbreviations

HDM, house dust mite; Der f, Dermatophagoides farinae; Der p, Dermatophagoides pteronyssinus; Der f 39, Group 39 allergen of Dermatophagoides farinae; Der p 39, Group 39 allergen of Dermatophagoides pteronyssinus; Der f 24, Group 24 allergen of Dermatophagoides farinae; Der p 24, Group 24 allergen of Dermatophagoides pteronyssinus; TnC, troponin C; IgE, immunoglobulin E; ELISA, enzyme-linked immunosorbent assay; IPTG, isopropyl-β-d-thiogalactopyranoside; HRP, horseradish peroxidase.

## Fundings

The present study was supported in part by research funding from the 10.13039/501100001809National Natural Science Foundation of China (grant no. 82071806), Guangdong Province (grant no. 2021A1515011140), and 10.13039/501100004791Shenzhen City (grant no. JCYJ20210324095004012 and JCYJ20190808155603545).

## Availability of data and materials

The datasets used and/or analyzed during the current study are available from the corresponding author on reasonable request.

## Author contributions

WYL, ZLC and BPZ performed experiments and interpreted results. WYL, ZLC and BPZ contributed to the data analysis. JJC and KJ supervised the projects and participated in experimental design and technical discussions. WYL and ZLC wrote the paper. JJC and KJ revised the manuscript.

## Ethics approval and consent to participate

Permission to conduct this study was obtained from the Ethics Committee of the First Affiliated Hospital of Guangzhou Medical College (No. 2012-51). Informed consent was obtained from all individual participants included in the study. All procedures involving human participants were in accordance with the ethical standards of the committee.

## Authors’ consent for publication

I confirm that each of the authors has reviewed this paper in its submitted form and approved submission for publication of this paper to the World Allergy Organization Journal.

## Declaration of competing interest

The authors declare no competing interests.
