# Supplementary material for: Identification of an immunodominant IgE epitope of Der p 39, a novel allergen of Dermatophagoides pteronyssinus
Source: World Allergy Organ J. 2022 May 6;15(5):100651. doi: 10.1016/j.waojou.2022.100651 (PMC9096144; doi:10.1016/j.waojou.2022.100651)
Supplement: Multimedia component 1 [file mmc1.docx]

**TABLE S1.** Demographic and clinical characteristics of HDM-allergic patients (P1-87) and 30 nonallergic individuals (NC1-30)

| **Patient no.** | **Age (years)** | | **Sex** | **HDM-related symptoms** | **Specific IgE to Der p (kUA/L)*** |
| --- | --- | --- | --- | --- | --- |
| P1 | | 24 | M | AR | 17.4 |
| P2 | | 18 | M | U | 9.21 |
| P3 | | 65 | M | BA | 3.49 |
| P4 | | 42 | F | AR | 12.3 |
| P5 | | 9 | M | U | 1.3 |
| P6 | | 13 | M | U | 16 |
| P7 | | 31 | M | U | 3.13 |
| P8 | | 24 | F | AR | 5.96 |
| P9 | | 42 | F | AR,AD | 7.09 |
| P10 | | 13 | M | BA | 16.2 |
| P11 | | 36 | F | AR | 5.73 |
| P12 | | 38 | F | AR | 11.4 |
| P13 | | 9 | F | AD,C | 9.5 |
| P14 | | 32 | F | AD | 4.12 |
| P15 | | 30 | F | E,U | 5.09 |
| P16 | | 44 | M | AR | 8.39 |
| P17 | | 28 | M | AR | 3.8 |
| P18 | | 16 | M | AR,U | 8.24 |
| P19 | | 60 | F | U | 2.66 |
| P20 | | 33 | F | AR | 5.91 |
| P21 | | 46 | F | AD,U | 6.04 |
| P22 | | 30 | M | AR,BA | 11.2 |
| P23 | | 57 | F | AD | 10.4 |
| P24 | | 8 | M | AR | 12.7 |
| P25 | | 22 | M | U | 5.56 |
| P26 | | 29 | F | AR | 3.42 |
| P27 | | 25 | F | BA | 1.2 |
| P28 | | 35 | M | AR | 17.4 |
| P29 | | 38 | F | E,U | 4.53 |
| P30 | | 35 | F | U | 5.57 |
| P31 | | 33 | M | BA | 9.96 |
| P32 | | 33 | F | AR,U | 5.06 |
| P33 | | 32 | F | AR,BA | 6.28 |
| P34 | | 19 | M | AR | 5.01 |
| P35 | | 39 | M | AR,BA | 4.74 |
| P36 | | 22 | M | AR,BA | 12 |
| P37 | | 49 | M | E | 7.44 |
| P38 | | 35 | M | AD | 9.57 |
|  | | | | | |

**TABLE S1.** (Continued)

| **Patient no.** | **Age (years)** | | **Sex** | **HDM-related symptoms** | **Specific IgE to Der p (kUA/L)*** |
| --- | --- | --- | --- | --- | --- |
| P39 | | 18 | F | U | 8.85 |
| P40 | | 19 | F | E | 5.74 |
| P41 | | 45 | M | AR | 13.5 |
| P42 | | 78 | F | P | 4.29 |
| P43 | | 23 | F | U | 11.4 |
| P44 | | 23 | F | U | 11.4 |
| P45 | | 32 | F | AD | 1.66 |
| P46 | | 58 | M | U | 2.71 |
| P47 | | 24 | F | AR | 1.11 |
| P48 | | 50 | F | AR,BA | 3.89 |
| P49 | | 28 | F | U | 1.45 |
| P50 | | 60 | F | AR,BA | 2.66 |
| P51 | | 60 | F | U | 2.66 |
| P52 | | 47 | M | AD | 1 |
| P53 | | 14 | F | AR,BA | 15.5 |
| P54 | | 29 | M | AR | 5.06 |
| P55 | | 32 | F | AR | 9.75 |
| P56 | | 5 | F | BA | 7.53 |
| P57 | | 52 | M | AR | 8.95 |
| P58 | | 25 | M | AR | 5.28 |
| P59 | | 28 | F | AD | 13.6 |
| P60 | | 42 | M | AR | 3.82 |
| P61 | | 12 | M | U | 4.13 |
| P62 | | 30 | M | AR | 13.1 |
| P63 | | 57 | F | AR | 44.1 |
| P64 | | 17 | F | AR,BA | 4.18 |
| P65 | | 20 | M | AR | 12.9 |
| P66 | | 4 | M | AR | 12.7 |
| P67 | | 47 | F | AR | 4.61 |
| P68 | | 28 | M | AR | 13.8 |
| P69 | | 43 | F | BA | 7.1 |
| P70 | | 50 | F | AR,BA | 16.3 |
| P71 | | 38 | M | AR,BA | 10.3 |
| P72 | | 14 | M | AR | 7.03 |
| P73 | | 51 | F | AR,BA | 15.9 |
| P74 | | 27 | F | AR | 16.6 |
| P75 | | 21 | M | AR,BA | 4.2 |
| P76 | | 31 | M | AR | 7.15 |
| P77 | | 13 | M | AR | 10.1 |
| P78 | | 28 | M | AR,BA | 12.6 |
| P79 | | 21 | F | AR,BA,C | 12.5 |

**TABLE S1.** (Continued)

| **Patient no.** | **Age (years)** | | **Sex** | **HDM-related symptoms** | **Specific IgE to Der p (kUA/L)*** |
| --- | --- | --- | --- | --- | --- |
| P80 | | 35 | M | AR | 11.5 |
| P81 | | 29 | F | AR, BA | 9.41 |
| P82 | | 30 | M | AR, C | 5.9 |
| P83 | | 40 | M | AR, BA | 23.9 |
| P84 | | 30 | M | AR | 12.8 |
| P85 | | 49 | M | AR, BA | 2.9 |
| P86 | | 24 | F | AR, BA | 4.8 |
| P87 | | 46 | F | BA | 0.15 |
| NC1 | | 11 | M | None | 0.00 |
| NC2 | | 43 | F | None | 0.00 |
| NC3 | | 29 | M | None | 0.00 |
| NC4 | | 23 | M | None | 0.00 |
| NC5 | | 58 | F | None | 0.00 |
| NC6 | | 31 | M | None | 0.00 |
| NC7 | | 9 | F | None | 0.00 |
| NC8 | | 54 | F | None | 0.00 |
| NC9 | | 30 | F | None | 0.00 |
| NC10 | | 35 | M | None | 0.00 |
| NC11 | | 26 | M | None | 0.00 |
| NC12 | | 5 | F | None | 0.00 |
| NC13 | | 15 | F | None | 0.00 |
| NC14 | | 40 | M | None | 0.00 |
| NC15 | | 48 | F | None | 0.00 |
| NC16 | | 51 | M | None | 0.00 |
| NC17 | | 33 | M | None | 0.00 |
| NC18 | | 62 | F | None | 0.00 |
| NC19 | | 43 | M | None | 0.00 |
| NC20 | | 15 | F | None | 0.00 |
| NC21 | | 50 | M | None | 0.00 |
| NC22 | | 44 | F | None | 0.00 |
| NC23 | | 58 | M | None | 0.00 |
| NC24 | | 32 | F | None | 0.00 |
| NC25 | | 41 | M | None | 0.00 |
| NC26 | | 17 | F | None | 0.00 |
| NC27 | | 37 | F | None | 0.00 |
| NC28 | | 49 | M | None | 0.00 |
| NC29 | | 27 | F | None | 0.00 |
| NC30 | | 16 | F | None | 0.00 |

*****Specific IgE antibodies to Der p were determined by ImmunoCAP (d1; Pharmacia Diagnostics, Uppsala, Sweden). HDM, house dust mite; Der p, *Dermatophagoides pteronyssinus*; F, female; M, male; AR, allergic rhinitis; AD, atopic dermatitis; BA bronchial asthma; C, conjunctivitis; U, urticaria; E, eczema; P, pruritus
